# A systematic review exploring the significance of measuring epicardial fat thickness in correlation to B-type natriuretic peptide levels as prognostic and diagnostic markers in patients with or at risk of heart failure

**DOI:** 10.1007/s10741-021-10160-3

**Published:** 2021-10-20

**Authors:** Thembeka A. Nyawo, Phiwayinkosi V. Dludla, Sithandiwe E. Mazibuko-Mbeje, Sinenhlanhla X. H. Mthembu, Tawanda M. Nyambuya, Bongani B. Nkambule, Hanél Sadie-Van Gijsen, Hans Strijdom, Carmen Pheiffer

**Affiliations:** 1 Biomedical Research and Innovation Platform, South African Medical Research Council, Tygerberg, 7505 South Africa; 2 Centre for Cardiometabolic Research in Africa (CARMA), Division of Medical Physiology, Faculty of Medicine and Health Sciences, Stellenbosch University, Tygerberg, 7505 South Africa; 3 Department of Biochemistry, Faculty of Natural and Agricultural Sciences, North West University, Mafikeng Campus, Mmabatho, 2735 South Africa; 4 Department of Health Sciences, Faculty of Health and Applied Sciences, Namibia University of Science and Technology, Windhoek, 9000 Namibia; 5 School of Laboratory Medicine and Medical Sciences, College of Health Sciences, University of KwaZulu-Natal, Durban, 4000 South Africa; 6 Department of Obstetrics and Gynaecology, University of Pretoria, Private Bag X169, Pretoria, 0001 South Africa

**Keywords:** Epicardial adipose tissue, B-type natriuretic peptide, Cardiovascular disease, Heart failure, Metabolic syndrome, Cardiac markers

## Abstract

Emerging evidence suggests that epicardial fat thickness (EFT) may be a critical feature to understand cardiac health and determine the risk of heart failure. The current review critically assesses and discusses evidence on the efficiency of measuring EFT, in comparison to the well-known markers B-type natriuretic peptide (BNP) and its N-terminal fragment pro-B-type natriuretic peptide (NT-proBNP), as a prognostic and diagnostic approach in individuals with or at risk of heart failure. A systematic approach was undertaken to search major databases, PubMed, Scopus, Google Scholar and the Cochrane library to identify studies that quantified EFT and serum BNP/NT-proBNP levels in individuals with or at risk of heart failure. Twelve studies met the inclusion criteria and a total of 1983 participants were included in this systematic review. Evidence shows a clear association between increased EFT and elevated BNP/NT-proBNP levels in individuals with metabolic disease and suggests that both methods can be used for heart failure diagnosis and prognosis. However, due to the broad spectrum of challenges linked with measuring EFT, BNP/Pro-BNP is the predominant method used for heart failure diagnosis and prognosis in clinical practice. Nonetheless, measuring EFT provides a powerful and reproducible diagnostic tool for risk stratification and heart failure diagnosis and prognosis. Importantly, measuring EFT proves valuable to validate BNP/NT-proBNP levels to predict heart failure, especially due to its non-invasive nature.

**Supplementary Information:**

The online version contains supplementary material available at 10.1007/s10741-021-10160-3.

## Introduction

Heart failure results from structural and functional defects in the myocardium which lead to impaired ventricular filling or ejection of blood into circulation. While the central element of heart failure may be ventricular dysfunction, other prominent defects include abnormalities in the pericardium, endocardium, as well as heart valves and major vessels [1, 2]. These defects result from singular or combinations of pathological mechanisms such as ischemia-related damage, abnormal ion handling, accelerated apoptosis, fibrosis or genetic mutations [3]. Clinically, heart failure diagnosis is based on assessment of signs and symptoms, blood tests including complete blood count, complete metabolic profile for serum electrolyte levels, fasting lipid profile, urinalysis and liver function tests [3]. Technological advances have provided more sensitive and specific heart failure laboratory tests, such as cardiac biomarkers, which consist of a wide range of biomolecules secreted by the myocardium in response to structural derangements. Notably, because of their diagnostic and prognostic value, these biomarkers can be useful for identifying individuals at increased risk of heart failure or monitoring patient response to therapeutic interventions [4].

Various biomarkers have been identified depending on their pathophysiological impact on cardiomyocytes. These include biomarkers of systemic inflammation, oxidative stress, myocardial stress, myocardial injury, neurohormones and renal function [3]. Natriuretic peptides, especially brain natriuretic peptides (BNP) and its N-terminal fragment (NT-proBNP), have been extensively used as biomarkers to detect acute heart failure [5–7]. They are currently commonly used biomarkers to detect myocardial strain. The peptide BNP is synthesized in cardiomyocytes as a pre-neurohormone, which is cleaved into a C-terminal fragment (BNP) and the biologically inactive N-terminal fragment (NT-proBNP) after release into circulation [8]. The synthesis and release of BNP is primarily regulated by ventricular stretch, and elevated levels of BNP have been reported in cases of left ventricular end diastolic pressure and pulmonary artery pressure [9]. Accumulating evidence suggests that increased secretion of BNP from overloaded left ventricles in patients with chronic heart failure may serve as a useful prognostic marker indicating hospital admission and discharge [10–12]. The clinical significance of BNP and NT-proBNP in heart failure diagnosis and prognosis is attributed to their increased sensitivity and high specificity to detect myocardial injury [13].

There is no difference in the predictive accuracy of BNP and NT-proBNP and both peptides independently predict heart failure outcome [8, 14], although NT-proBNP has been demonstrated to have a longer half-life than BNP [3]. The diagnostic value of BNP and NT-proBNP has been well established, with several studies showing a strong correlation between elevated levels of BNP and NT-proBNP in hospitalised patients with heart failure and risk of death [15]. Although there is evidence to support using serum BNP and NT-proBNP levels as adjunctive markers to define the progression of heart failure, limitations such as stratifying heterogeneous patient groups or effectively identifying those presenting with heart failure with preserved ejection fraction are persistently mentioned [16, 17]. Elevated BNP/NT-pro BNP levels have been reported in circumstances other than heart failure, for example, renal failure, lung disease with right-sided failure, acute coronary syndrome and acute large pulmonary embolism. These limitations highlight the importance of using additional parameters for heart failure diagnosis and prognosis.

In recent years, a growing number of studies have provided evidence that epicardial fat thickness (EFT) plays an important role in the development and progression of heart failure. Echocardiography of EFT has contributed to understanding the relationship between epicardial adipose tissue (EAT) and the underlying myocardial defects that confer heart failure [18]. Lipotoxicity, inflammation and oxidative stress are major factors that contribute to the early onset of metabolic disorders. These pathophysiological mechanisms have been associated with increased EFT and instigate intrinsic myocardial dysfunction [3]. EAT mediates pathophysiological processes of heart failure by regulating adipogenesis, insulin resistance, the renin angiotensin aldosterone system (RAAS), cardiac remodelling and cardiac output [18]. Although clinical detection of these early myocardial alterations remains a challenge, measuring EFT may predict the presence of early myocardial derangements. Studies have shown that differences in EFT are associated with the presence and severity of cardiometabolic diseases. It has been reported that echocardiographic EFT is linked with visceral adiposity [19, 20], while other studies have shown a linear correlation between EFT, left ventricular mass and severity of coronary artery disease (CAD) [20]. Increased EFT in heart failure patients with established metabolic disturbances such as diabetes have also been reported [21].

Taken together, these findings indicate that measuring both EFT and BNP/NT-proBNP may offer increased sensitivity and predictive ability of clinical outcomes, including mortality and rehospitalization. Consequently, measuring EFT, in combination with assessing serum biomarkers like BNP/NT-proBNP, may serve as potential prognostic tools in heart failure patients and aid to identify patients at increased risk of heart failure. The present systematic review aims to critically assess and discuss studies that measured EFT and serum BNP/NT-proBNP levels as a diagnostic and prognostic approach in individuals with or at risk of heart failure. Importantly, this review will report on the efficiency of determining the levels of BNP/NT-proBNP in correlation with EFT to classify individuals with or at risk of heart failure.

## Methodology

### Search strategy and study selection

A search of articles indexed in electronic databases PubMed, Scopus, Google Scholar and Cochrane library between date of inception and 10 February 2021 was conducted. The search terms included the medical subject heading (MeSH) term “B-type natriuretic peptide” and keywords “epicardial adipose tissue” “epicardial fat thickness” as well as corresponding terms. References were managed and duplicate studies removed in Mendeley (version 1.1.18). The titles and abstracts of the articles from the electronic search outputs were screened independently by two reviewers (TAN and TMN) to identify eligible studies, while a third reviewer (PVD) was consulted for adjudication when required. Full-text copies of eligible articles were retrieved and reviewed by two independent reviewers (TAN and TMN) for inclusion, while discrepancies were resolved by a third reviewer (PVD).

### Inclusion criteria

The systematic review included studies reporting EFT and serum levels of BNP/NT-proBNP in individuals with or at risk of heart failure. Only articles reporting primary findings were included, while reviews and letters were excluded. Review articles were screened to identify studies that may have been missed using our search strategy. This systematic review was conducted to answer the following questions:

- Question 1: Are EFT and BNP/NT-proBNP levels diagnostic or prognostic markers of heart failure?
- Question 2: Is EFT and BNP/NT-proBNP differentially regulated in individuals with or at-risk of heart failure?

This was achieved using the following:

- Participants: Individuals with heart failure or individuals with an increased risk of developing heart failure such as those with cardiometabolic diseases.
- Exposure: No intervention was used in this study.
- Comparator: Individuals without overt heart failure and/or at risk of heart failure.
- Outcome: EFT and BNP/NT-proBNP levels.

### Data extraction and quality assessment

Data were independently extracted and tabulated in Microsoft Excel based on study details (authors, date of publication, study title and design, sample size and main findings), characteristics of population (sex, age, etc.) and measurements of EFT and BNP/NT-proBNP. Two reviewers (TAN and SXH) independently appraised the study quality and risk of bias using the Newcastle–Ottawa Scale, which is appropriate for cross-sectional and case–control studies [22]. The checklist evaluates three quality parameters: selection, comparability and outcomes, which are divided into eight specific items that can be scored from one up to two points (supplementary file. S1). Disagreements between the reviewers were resolved by consulting a third reviewer (PVD). The inter-rater reliability of the scoring process was assessed using the Cohen’s Kappa (*k*) statistics tool. The kappa scores can range from − 1 to + 1, where 0 represents the amount of agreement that can be expected from random chance, and 1 represents perfect agreement between the reviewers. Kappa values ≤ 0 indicates no agreement, 0.01–0.20 as none to slight, 0.21–0.40 as fair, 0.41– 0.60 as moderate, 0.61–0.80 as substantial and 0.81–1.00 as almost perfect agreement.

## Results

### Selected studies

**Fig. 1:**
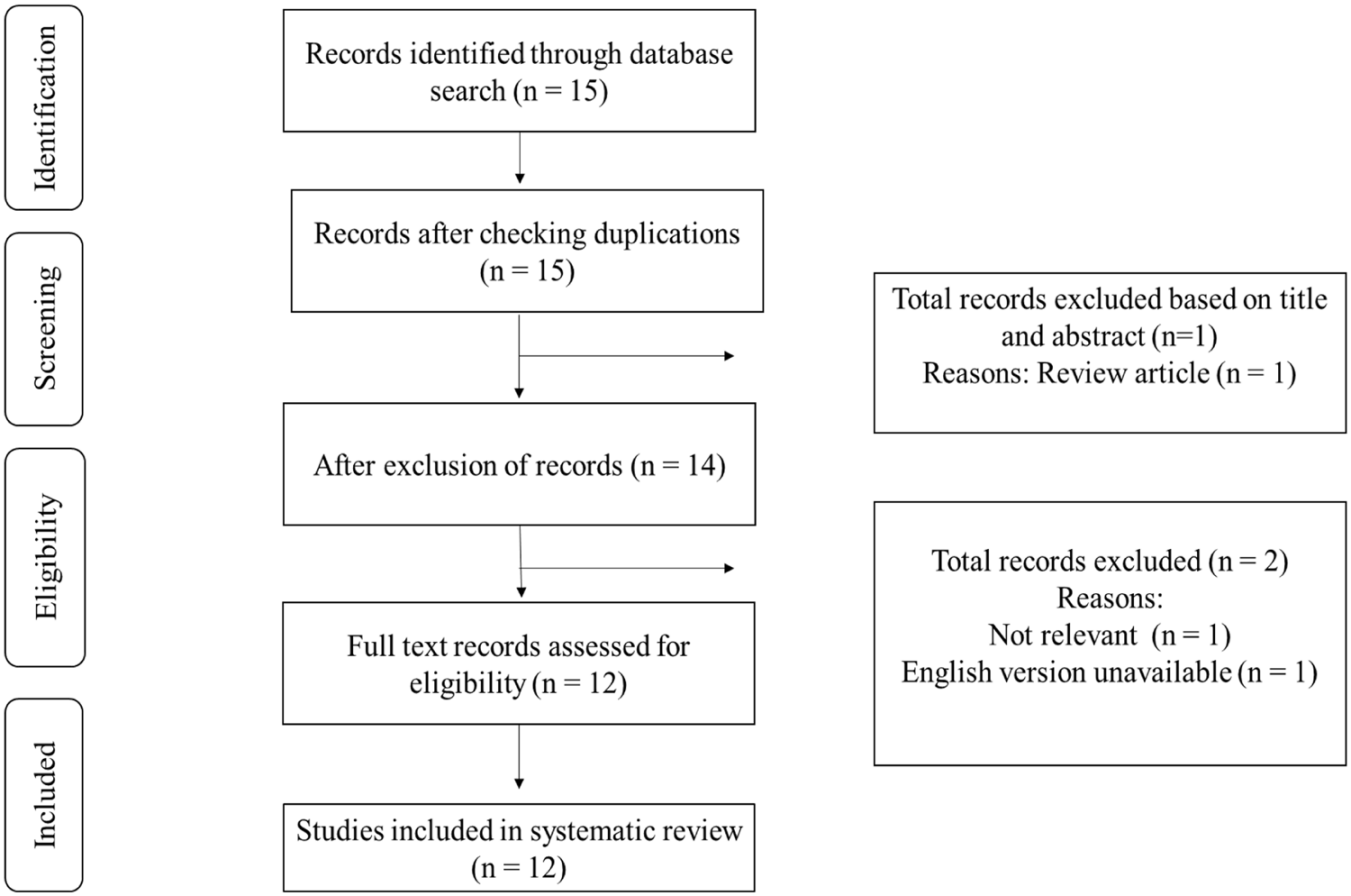
Diagrammatic representation of study selection process. Briefly, a total of 15 studies were identified from the search strategy, and of these, 12 met the inclusion criteria and measured epicardial fat thickness (EFT) and the serum levels of B-type natriuretic peptide in patients with heart failure or individuals at risk of developing heart failure

A total of 15 studies were identified from the search strategy, and of these, 12 studies reported on the association between EFT and serum levels of BNP/NT-proBNP in patients with heart failure or individuals at risk of heart failure. Studies were excluded if they were reviews [23], not available in English [24] or measured peri-coronary adipose tissue instead of EAT [25], as represented on the flow diagram (Fig. 1). As a result, a total of 12 studies, published between 2010 and 2020, met the inclusion criteria (overall agreement: 93.75%, kappa: 0.82) and are discussed in this review.

### Characteristic features of included studies

Of the 12 included articles, six studies reported on EFT and BNP/NT-proBNP levels in heart failure patients (*n* = 1071) and six studies reported on EFT and BNP/NT-proBNP levels in patients at risk of heart failure (*n* = 912). Participants at risk for heart failure had Cushing’s syndrome, obesity, coronary artery disease (CAD), non-ischemic dilated cardiomyopathy (NIDCP), acute ischemic stroke (AIS) and systematic sclerosis (SSc). Five studies were conducted in Turkey and one each in Greece, Norway, China, Spain, Netherland, the USA, and Italy. The study designs were cross-sectional (*n* = 1) and case control (*n* = 11). Ten studies were conducted in adults with an age range of 49.4 ± 8.4 to 71.4 ± 11 years, while two studies were conducted in children with ages ranging between 10.46 ± 2.38 and 14.3 ± 1.7 years.

### Quality assessment of included studies

The quality of the 12 included studies ranged from unsatisfactory to good with numerical scores ranging from 6 to 10 and a median score of 8. Two studies scored as very good, seven studies scored as good, two studies were scored as satisfactory and one study scored unsatisfactory. The inter-rater reliability of all the domains was assessed using Cohen’s kappa. The agreement was scored as substantial agreement for the selection of participants with 75% agreement (*k* = 0.50; 95% CI: − 0.48, 1.00), perfect agreement for comparability with 100% agreement (*k* = 1.00; 95%) and an agreement equal to chance for the outcome with 50% agreement (*k* = 0; 95% CI: − 1.00, 1.00). An overview of the quality assessment scores can be found in the supplementary file S2.

### Qualitative synthesis

#### Evidence of the link between BNP/NT-proBNP levels and epicardial fat thickness in patients with heart failure

Studies reporting on levels of BNP and EFT in patients with clinically diagnosed heart failure are listed in Table 1. Here, BNP/NT-proBNP levels and/or EFT are reported in patients with heart failure either in response to nutritional status, with other comorbidities such as type 2 diabetes (T2D), or to stratify those with reduced or preserved ejection fraction.

**Table 1:** An overview of studies reporting on the correlation between epicardial fat and B-type natriuretic peptide levels in individuals with heart failure

Author	Study design	Participants	N	Age (years)	EFT (mm)	BNP (pg/ml)	Sex (male)	Country	Main findings	Quality assessment
[26]	Case-control	Heart Failure	57	68 ± 12	3.9	516000	96 (79%)	Greece	Epicardial fat thickness (EFT) did not differ in patients with heart failure compared to controls, while a negative correlation between EFT and B-type natriuretic peptide (BNP) serum levels in the heart failure group was observed	Good
		Control	64	64 ± 11	3.8	79000				
[27]	Case-control	Heart failure	30	57.0 ± 15.8	Not reported	2871	40 (67%)	Norway	Patients in the heart failure group exhibited higher levels of NT-proBNP, compared to the control group, which was associated with reduced systolic cardiac function with lower left ventricular ejection fraction	Good
		Control	30	59 ± 17.7		546				
[28]	Case-control	Heart failure	110	68 ± 8	Not reported	2498	92 (58%)	China	Patients with heart failure had increased levels of BNP, consistent with increased C1q and tumour necrosis factor-related protein 1 (CTRP1) levels in the plasma and EAT, compared to controls	Good
		Control	50	67 ± 5		14				
[29]	Case–control	Normal nutrition	31	67.2 ± 10.9	Not reported	2669.4	61.3	Spain	There was a strong association between BNP levels, upregulated EAT adiponectin levels, and failing nutritional status, where heart failure patients with worse malnutrition had the highest BNP levels	Very good
		Mild malnutrition	35	67.1 ± 12.5		4167				
		Moderate to severe malnutrition	8	72.0 ± 7.96		9231				
[30]	Case–control	Heart failure	64	70 ± 10.7	107#	885	53 (42%)	The Netherlands	EFT was significantly higher in heart failure patients compared to controls.	Good
		Controls	20	66 ± 5.5	77#	Not reported				
[31]	Case-control	HFrEF	113	65 (60.0–70.0)	4.9	2,748	530 (92%)	USA	Patients with HFrEF and HFpEF had higher NT-proBNP levels compared to the control group. In addition to other measures of adiposity, EFT was independently associated with increased NT-proBNP levels irrespective of heart failure status	Very good
		HFpEF	92	64 (59.0–71.0)	4.8	486				
		Controls	367	63 (57.0–68.8)*	4.8	325				

Age is indicated as mean ± SD or *median and interquartile range. #ml/m2 *HFrEF* heart failure with reduced ejection fraction, *HFpEF* heart failure with preserved ejection fraction, *EFT* epicardial fat thickness, *BNP* brain natriuretic peptide, *NT-proBNP* N-terminal proBNP

Three studies reported on EFT and BNP/NT-proBNP in patients with heart failure. Karayannis and colleagues showed that EFT did not differ in patients with heart failure compared to controls, while EFT was negatively correlated with serum BNP levels [26]. In contrast, van Woerden et al. observed higher EFT in heart failure patients compared to controls [30]. Selvaraj et al. observed no difference in EFT and higher levels of NT-proBNP in heart failure patients compared to controls [31]. Fosshaug and co-workers reported on an altered metabolic profile that was consistent with an aberrant inflammatory status within the EAT of patients with heart failure when compared to controls [27]. Interestingly, these inflammatory effects were associated with reduced systolic cardiac function and lower left ventricular ejection fraction, suggesting that established metabolic complications such as inflammation are strong predictors of myocardial dysfunction, as previously discussed elsewhere [32]. Indeed, this effect is further confirmed by Yang and colleagues, who reported that patients with congestive heart failure displayed elevated levels of BNP, complement component 1q (C1q) and tumour necrosis factor-related protein 1 (CTRP1) in plasma and EAT when compared to controls [28]. These results are in line with others indicating that CTRP1, an adipose tissue-derived adiponectin family paralog, is associated with both increased body mass index and pathogenesis of CAD [32, 33]. In line with this work, Agra and co-workers showed a strong association between high BNP levels, upregulated EAT adiponectin expression, and poor nutritional status, where heart failure patients with worsening degrees of malnutrition had the highest BNP levels [29]. Patients with established heart failure appeared to display elevated levels of BNP/NT-proBNP and high EFT indicating that both markers can potentially be used in heart failure diagnosis and prognosis.

#### Evidence on the link between BNP levels and epicardial adipose tissue thickness in patients at risk of heart failure

Currently, it is understood that a variety of conditions can impair cardiac function, leading to increased cardiovascular disease (CVD) risk and subsequent heart failure. Thus, it remains essential to identify potential diagnostic features such as EFT, in conjunction with elevated BNP levels to detect risk of heart failure in patients with comorbidities. Six studies reported EFT and BNP/NT-proBNP levels in patients with diseases associated with metabolic dysfunction (Table 2).

**Table 2:** An overview of studies reporting on the correlation between epicardial fat thickness and B-type natriuretic peptide levels in individuals at risk of heart failure

Author	Study design	Participants	N	Age (years)	EFT (mm)	BNP (pg/ml)	Sex (male %)	Country	Main findings	Quality assessment
[34]	Case–control	Cushing’s syndrome	23	14.3 ± 1.7	7.1	109.1	None	Italy	In comparison to the control group, patients with Cushing’s syndrome had significantly higher epicardial fat thickness (EFT) and N-terminal pro-B-type natriuretic (NT-proBNP) levels, indicating a positive correlation. This underlines a significantly increased cardiovascular disease (CVD) risk in patients with Cushing’s disease in younger females	Good
		Controls	23	14.9 ± 1.5	2.6	53.8				
[35]	Case–control	Obese	50	10.4 ± 2.3	5.6	109.3	Unspecified	Turkey	Obese children showed significantly higher NT-proBNP and EFT levels compared to non-obese controls, indicating that obese children are at increased risk of developing cardiovascular disease and subsequent heart failure. However, no association between these markers and left ventricular systolic and diastolic functions were observed	Good
		Controls	20	10.1 ± 3.4	3.0	52.00				
[36]	Cross-sectional	CAD patients with low EFT		60.7 ± 10.9	4.7	128.9	286 (65%)	Turkey	EFT is positively correlated with NT-proBNP serum levels in patients with stable coronary artery disease (CAD)	Unsatisfactory
		CAD patients with high EFT 439$		63.7 ± 10.2	6.5	251.6				
[37]	Case–control	Non-ischemic dilated cardiomyopathy (NICMP)	93	49.9 ± 13.9	4.1	247000	87 (66%)	Turkey	Patients with NICMP have significantly decreased EFT compared to controls. In NICMP patients, EFT correlated inversely with BNP and predicts impaired cardiomyocyte function indicating the severity of heart failure in NICMP	Satisfactory
		Controls	38	51.1 ± 10.0	6.1	20000				
[38]	Case–control	Acute Ischemic Stroke (AIS)	61	71.4 ± 11	4.8	1 327	69 (48%)	Turkey	EFT is increased in AIS patients and correlates positively with NT-proBNP concentrations and aortic stiffness. EFT and NT-proBNP levels can provide information on arterial function in patients with AIS	Satisfactory
		Controls	82	68.6 ± 8	3.8	203				
[39]	Case–control	Systemic Sclerosis (SSc)	47	52.1 ± 12.4	6	111#	8 (10%)	Turkey	EFT was significantly increased in patients with SSc compared to the control group. Elevated BNP levels indicated a link between BNP and EFT in SSc patients without overt cardiovascular disease	Good
		Controls	36	49.4 ± 8.4	5	70				

Age is indicated as mean ± SD or *median and interquartile range. #mg/dl $total sample *HFrEF* heart failure with reduced ejection fraction, *HFpEF* heart failure with preserved ejection fraction, *EFT* epicardial fat thickness, *NT-proBNP* N-terminal-proBNP

Bassareo and colleagues demonstrated that patients with Cushing’s syndrome may present with an increased risk of heart failure as they display significantly higher EFT, which correlates with elevated NT-proBNP levels [34]. This remains essential to identify and classify early since patients with Cushing syndrome, a condition that is associated with obesity [40], may easily develop dilated cardiomyopathy and heart failure, with reduced ejection fraction [41]. However, in obese children, Saritas and colleagues did not observe any statistical difference in the levels of NT-proBNP in relation to the left ventricular systolic or diastolic functions, including carotid intima-media thickness, and EFT [35]. These findings suggest that elevated levels of NT-proBNP or EFT may not indicate pathological changes related to heart failure during the early development of obesity. Similarly, Börekçi and co-workers confirmed that EFT is positively correlated with NT-proBNP serum levels in patients with stable CAD [36]. Intriguingly, in a rare case, Tabakci and co-workers demonstrated that although BNP levels remained relatively high, patients with non-ischemic dilated cardiomyopathy presented with markedly reduced EFT in comparison to controls [37]. This suggests that measuring the combination of both EFT and BNP may be a better tool to unravel the different forms of heart failure, especially non-ischemic dilated cardiomyopathy. In another example, unlike in non-ischemic dilated cardiomyopathy, EFT is relatively high in patients with acute ischemic stroke, and correlates positively with NT-proBNP concentrations and aortic stiffness [38]. Karadag and colleagues reported that both EFT and BNP levels were significantly increased in patients with systemic sclerosis compared to the control group [31]. This evidence suggests that EFT and increased levels of BNP can easily identify patients with overt CVD, especially those with ischemic heart disease, while when used in combination, these prognostic markers can be used to stratify patients at risk of developing heart failure.

## Discussion

Early detection of clinical deterioration is a critical component of heart failure management to facilitate the initiation of appropriate and effective therapeutic strategies. Heart failure is a chronic and progressive multifaceted disorder which prompts inflammation and metabolic disturbances [41]. Cardiac biomarkers, which are classified according to the pathophysiological insults they exert on cardiomyocytes, have been applied to predict the clinical course of heart failure. Chronic overnutrition increases susceptibility to developing obesity and CVD, which, when left untreated, leads to heart failure. Patients with metabolic syndrome typically present with classical features of obesity such as adipose tissue expansion and increased EFT [42].

Although EAT may exert cardioprotective effects, through the secretion of anti-inflammatory adipokines and supplying energy in the form of triglycerides, an enhanced myocardial lipid supply and its oxidative capacity cause detrimental effects on the underlying myocardium [43, 44]. Fatty acid accumulation intensifies proinflammatory activity, including the increased expression of inflammatory cytokines. In agreement, some of the evidence included in this review show that enhanced EAT correlates with proinflammatory markers such as C1q, C-reactive protein and CTRP1 in patients with heart failure [26, 28]. These devastating effects associated with EAT expansion can directly lead to ventricular expansion that causes BNP release [45]. In addition to their significance in heart failure diagnosis, BNP levels provide additional prognostic information beyond the classical CVD risk factors and have also been shown to predict heart failure mortality independent of age, previous myocardial infarction and altered left ventricular ejection fraction [3]. The prognostic value of NT-proBNP has been shown in previous studies, where NT-proBNP levels were identified as independent predictors of mortality in hospitalized patients with heart failure [12, 46]. Also, NT-proBNP has been consistently correlated with elevated risk for mortality and/or rehospitalization for heart failure in patients with severe congestive heart failure [47]. Despite the available evidence of the use NT-proBNP as a prognostic marker, literature has also reported circumstances of elevated BNP levels in the absence of heart failure [10]. Moreover, BNP/NT-proBNP levels may vary between different fluid sample sources, where NT-proBNP levels were significantly lower in serum compared to pericardial fluid levels in heart failure patients with impaired left ventricular systolic function [48]. This therefore highlights the importance of evaluating additional biomarkers to improve heart failure diagnosis. Thus, measuring EFT can offer additional benefits for the following reasons: (1) EFT is an independent predictor of heart failure and is proposed as a heart failure biomarker, (2) a strong correlation between BNP/NT-proBNP levels and EFT already exists and (3) non-invasive nature of measuring EFT. In our study, we show that BNP/NT-proBNP levels as well as EFT are significantly elevated in heart failure patients, suggesting that these markers can be used concomitantly for heart failure diagnosis and prognosis.

Furthermore, evidence from the current review highlights a clear correlation between increased EFT and elevated levels of BNP/NT-proBNP in individuals with an increased risk of heart failure, particularly in the presence of illnesses such as CAD. Similar observations were identified in patients with systemic sclerosis [39], a condition consistent with overt CVD-related complications such as fibrosis, myocarditis, pulmonary hypertension and blood vessel abnormalities [49–51]. Interestingly, patients with AIS also displayed increased EFT, which was positively correlated with NT-proBNP levels [40]. This data provide evidence that even in the absence of overt heart failure, patients with other clinical conditions such as obesity, CAD, AIS and Cushing’s disease exhibit some of the phenotypes associated with heart failure, particularly increased EFT that usually correlates with elevated BNP/NT-proBNP levels. These findings show a strong correlation between EFT and BNP/NT-proBNP levels and support their potential to stratify individuals at increased risk of developing heart failure.

## Conclusion and future perspective

**Fig. 2:**
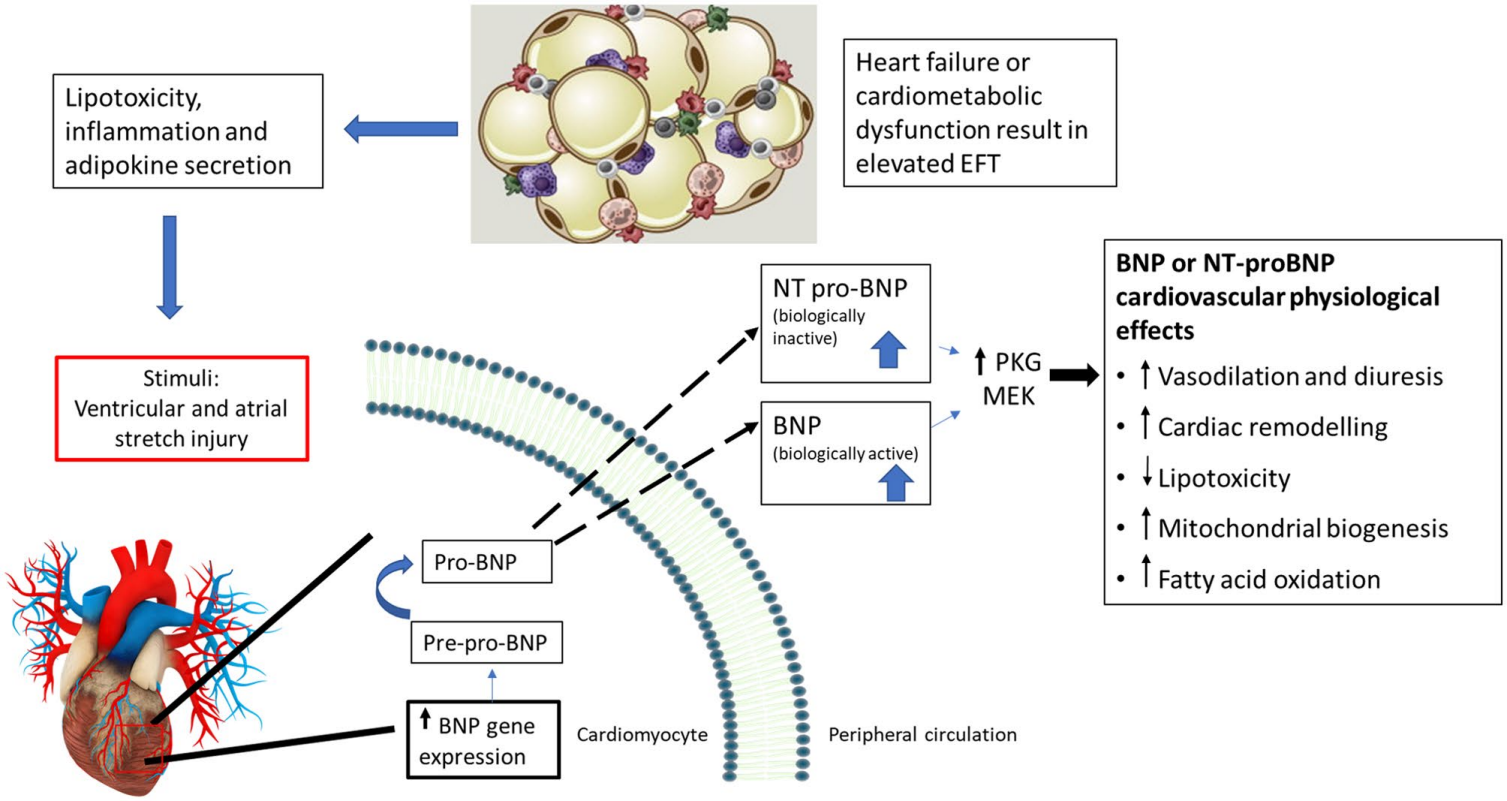
Epicardial fat expansion in the state of heart failure and/or in cardiometabolic dysfunction contributes to the development and progression of heart failure through multiple pathophysiological mechanisms including inflammation and adipokine secretion. These factors ultimately result in ventricular and atrial stretch, which in turn instigate the secretion of the widely used cardiac biomarker BNP/NT-proBNP. Despite the predominant use of BNP/NT-proBNP in heart failure diagnosis and prognosis, limitations to its use have been reported. In the current review, we highlight the association between EFT and BNP/NT-proBNP and how both parameters can potentially be used to add value to heart failure diagnosis and prognosis. PKG, Protein kinase G; MEK, mitogen-activated protein kinase

The summarized evidence suggests that measuring both EFT and BNP/NT-proBNP levels can be useful to classify patients with or at increased risk of heart failure. Currently, measuring BNP/NT-proBNP levels is the predominant method for heart failure prognosis and diagnosis in clinical practice due to the broad spectrum of challenges associated with EFT measurement. These challenges include difficulties in differentiating between epicardial fat and pericardial fat, undefined cut-off values for EFT which varies across disease spectrum, high costs of EFT measuring techniques compared to BNP/NT-proBNP measurements, the unavailability of resources to measure EFT in low-income countries and ethnic differences in EFT [19] (Fig. 2). Despite these limiting factors, measuring EFT provides a powerful and reproducible diagnostic tool for risk stratification and heart failure diagnosis and prognosis. Importantly, measuring EFT proves valuable to validate BNP/NT-proBNP levels to predict heart failure, especially due to its non-invasive nature.

## Supplementary Information

Below is the link to the electronic supplementary material.

Supplementary file1 (PDF 148 KB)

Supplementary file2 (XLSX 12 KB)

## Data Availability

Data related to search strategy, study selection and extraction items will be made available upon request after the manuscript is published.

## Abbreviations

CVD: Cardiovascular disease
BNP: Brain natriuretic peptide
NT-proBNP: N-terminal fragment brain natriuretic peptide
EFT: Epicardial fat thickness
EAT: Epicardial adipose tissue
CAD: Coronary artery disease
NICDP: Non-ischemic dilated cardiomyopathy
AIS: Acute ischemic stroke
SSc: Systematic sclerosis
T2D: Type 2 diabetes
HFrEF: Heart failure with reduced ejection fraction
HFpEF: Heart failure with preserved ejection fraction.
